# Tumor-Specific Monomethyl Auristatin E (MMAE) Prodrug Nanoparticles for Safe and Effective Chemotherapy

**DOI:** 10.3390/pharmaceutics14102131

**Published:** 2022-10-07

**Authors:** Hanhee Cho, Man Kyu Shim, Yujeong Moon, Sukyung Song, Jinseong Kim, Jiwoong Choi, Jeongrae Kim, Youngjoo Lee, Jung Yeon Park, Yongju Kim, Cheol-Hee Ahn, Mi Ra Kim, Hong Yeol Yoon, Kwangmeyung Kim

**Affiliations:** 1 Department of Materials Science and Engineering, Seoul National University, Seoul 08826, Korea; 2 Biomedical Research Division, Korea Institute of Science and Technology (KIST), Seoul 02792, Korea; 3 KU-KIST Graduate School of Converging Science and Technology, Korea University, Seoul 02841, Korea; 4 Department of Integrative Energy Engineering, KU-KIST Graduate School of Converging Science and Technology, Korea University, Seoul 02841, Korea; 5 Department of Otorhinolaryngology-Head and Neck Surgery, Haeundae Paik Hospital, College of Medicine, Inje University, Busan 48108, Korea; 6 Graduate School of Pharmaceutical Sciences, College of Pharmacy, Ewha Womans University, Seoul 03760, Korea

**Keywords:** prodrug, monomethyl auristatin E, nanoparticle, targeted therapy, chemotherapy

## Abstract

A prodrug is bioreversible medication that is specifically converted to the active drugs by enzymes overexpressed in the tumor microenvironment, which can considerably reduce the chemotherapy-induced side effects. However, prodrug strategies usually have low antitumor efficacy compared to free drugs by delayed drug release. This is because they need time to be activated by enzymatic cleavage and they also cannot be fully recovered to the active drugs. Therefore, highly potent anticancer drug should be considered to expect a sufficient antitumor efficacy. Herein, we propose tumor-specific monomethyl auristatin E (MMAE) prodrug nanoparticles for safe and effective chemotherapy. The cathepsin B-specific cleavable FRRG peptide and MMAE are chemically conjugated via one-step simple synthetic chemistry. The resulting FRRG-MMAE molecules form stable nanoparticles without any additional carrier materials by hydrophobic interaction-derived aggregations. The FRRG-MMAE nanoparticles efficiently accumulate within the tumor tissues owing to the enhanced permeability and retention (EPR) effect and inhibit the tubulin polymerization by releasing free MMAE in the cathepsin B-overexpressed tumor cells. In contrast, FRRG-MMAE nanoparticles maintain a non-toxic inactive state in the normal tissues owing to innately low cathepsin B expression, thereby reducing MMAE-related severe toxicity. Collectively, this study provides a promising approach for safe and effective chemotherapy via MMAE-based prodrug nanoparticles, which may open new avenues for advanced drug design for translational nanomedicine.

## 1. Introduction

Chemotherapy is still the most common approach for anticancer treatment owing to its considerable antitumor efficacy by high sensitivity in the broad spectrum of cancer types [1]. However, anticancer drugs are often accompanied by severe side effects during treatment because of their low tumor selectivity, which restricts drug dosage in vivo, resulting in treatment failure by limiting the tumors from being exposed to sufficient drug concentrations [2,3]. As a promising approach to improve the tumor selectivity of anticancer drugs, the prodrug is a bioreversible medication that is specifically converted to the active drug by chemical or enzymatic transformation in the tumor microenvironment, which can considerably reduce the chemotherapy-induced side effects [4,5]. Importantly, tumor tissues show significantly different characteristics in comparison to normal tissues, including high reactive oxygen species (ROS), elevated/low pH, and hypoxia condition [6]. In particular, the overexpression of several enzymes, including cathepsins, caspases, and matrix metalloproteinases (MMPs), is observed within the tumor microenvironment compared to normal tissues [7,8,9,10]. Thus, designed prodrugs releasing active drugs selectively by those overexpressed enzymes in the tumor microenvironment have been proposed to increase the safety of chemotherapy [11,12,13]. However, prodrug strategies usually have low antitumor efficacy compared to parent drugs by delayed drug release because they need time to be activated by enzymatic cleavage and also cannot be fully recovered to the active drugs [14]. Therefore, highly potent anticancer drug should be considered for development utilizing the prodrug system to expect a sufficient antitumor efficacy for tumor treatment.

Monomethyl auristatin E (MMAE), a synthetic analog of the natural product dolastatin 10, is a potent antimitotic agent that inhibits tubulin polymerization [15]. Even with the 100–1000 times more potent antitumor efficacy than doxorubicin, the clinical use of MMAE has been strictly hindered owing to the severe toxicity [3]. Hence, many researchers have developed antibody-drug conjugates (ADCs) integrating prodrug system by using MMAE; this is to reduce the severe toxicity of MMAE via prodrug strategy, and to further enhance the tumor selectivity by antibody-mediated active targeting against receptors specifically overexpressed in tumor cells compared to normal cells [16,17]. For instance, cAC10-vcMMAE, constructed with anti-CD30 monoclonal antibody (cAC10), cathepsin B-specific cleavable valine-citrulline (VC) dipeptide linker, and MMAE, has shown highly selective therapy for the tumor treatment with minimized toxicity [18]. However, ADCs typically carry one to four of anticancer drug molecules per antibody, and thus drug loading capacity is determined to be 1–4% in terms of molecular weight, which limit the antitumor efficacy. In addition, their complex synthetic chemistry hindering industrial-scale manufacturing is a formidable challenge for clinical translation. In addition, there were also several approaches to prepare MMAE modified with cytokines or peptides for superior antitumor efficacy, but their clinical use was strictly hindered owing to severe MMAE-related toxicities [19,20,21].

**Figure 1:**
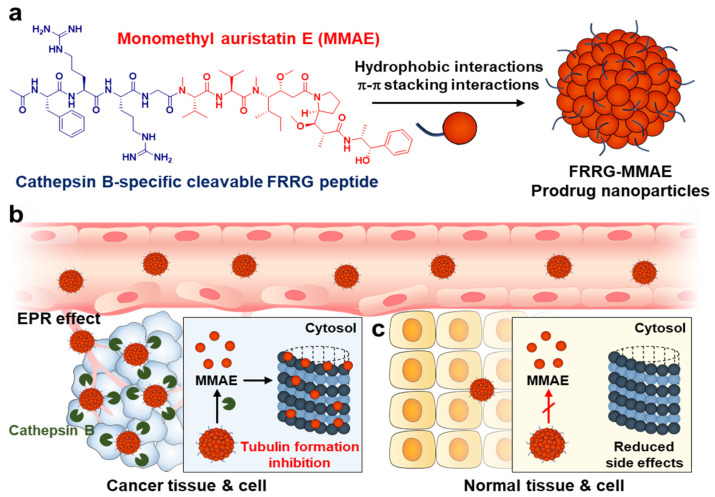
(**a**) The FRRG-MMAE is prepared by conjugating MMAE to the cathepsin B-cleavable FRRG (*Phe-Arg-Arg-Gly*) peptide. The FRRG-MMAE self-assembled into stable nanoparticles without any additional carrier materials by intermolecular hydrophobic interactions. (**b**) When the FRRG-MMAE nanoparticles are intravenously injected into the breast tumor models, they efficiently accumulate within the tumor tissues owing to the enhanced permeability and retention (EPR) effect and specifically release free MMAE by cathepsin B overexpressed in tumor cells. The released MMAE inhibits the tubulin polymerization in the tumor cells, resulting in significant cell death. (**c**) In contrast, FRRG-MMAE nanoparticles, which are non-specifically accumulated in the normal tissues, maintain inactive state owing to the innately low cathepsin B expression in the normal cells, minimizing the MMAE-related severe toxicity.

Here, we propose tumor-specific MMAE prodrug nanoparticles for safe and effective chemotherapy. The MMAE is chemically conjugated with the cathepsin B-cleavable FRRG (*Phe-Arg-Arg-Gly*) peptide via simple one step synthetic chemistry, resulting in FRRG-MMAE (Figure 1a). The FRRG sequence is a substrate for the cathepsin B, which is a promising cancer biomarker overexpressed specifically in tumor cells compared to normal cells. Moreover, they efficiently release the active drug by sequential cleavage mechanisms by which -RR- sequences are firstly cleaved by cathepsin B, and the released glycine (G)-conjugated drug is subsequently metabolized into the free drug by intracellular proteases [22,23,24,25,26,27,28]. The target enzyme specificity of FRRG sequence was confirmed by assessing reduced antitumor efficacy of the doxorubicin when they were conjugated with scrambled FGRG peptide instead of FRRG peptide. FGRG-conjugated doxorubicin formed stable nanoparticles similar with FRRG-conjugated doxorubicin, but they showed no cytotoxicity owing to the absence of enzyme-specific cleavage mechanism. Most importantly, FRRG-MMAE self-assembled into stable nanoparticles without any additional carrier materials by hydrophobic interaction-derived aggregations [29]. This novel carrier-free nanoparticle system has ultra-high drug loading capacity (>50%) and favorable synthetic protocol for large-scale production, and reduces the potential toxicity by carrier materials, compared to conventional nanoparticles that physically encapsulates the anticancer drugs. When the FRRG-MMAE nanoparticles are intravenously injected into the breast tumor-bearing mice, they efficiently accumulate within the tumor tissues by enhanced permeability and retention (EPR) effect and release free MMAE by cathepsin B overexpressed in tumor cells (Figure 1b). The released MMAE molecules inhibit the tubulin polymerization in the tumor cells, resulting in significant cell death. In contrast, FRRG-MMAE nanoparticles, which are non-specifically accumulated in the normal tissues, maintain an inactive state owing to the innately low cathepsin B expression in the normal cells, minimizing the MMAE-related severe toxicity (Figure 1c). In this study, the physicochemical characterization of FRRG-MMAE nanoparticles, and their intracellular behavior and cytotoxicity in tumor or normal cells are investigated. In addition, the safe and effective chemotherapy by FRRG-MMAE nanoparticles is evaluated in the breast tumor-bearing mice.

## 2. Materials and Methods

### 2.1. Reagents

N-terminal acylated FRRG (Ac-*Phe-Arg-Arg-Gly*-COOH) and FRRG (NH_2_-*Phe-Arg-Arg-Gly*-COOH) peptides were purchased from Peptron (Daejeon, Korea). Monomethyl auristatin E (MMAE) was purchased from MedChemExpress (Monmouth Junction, NJ, USA) Dimethylformamide (DMF), 1-ethyl-3-(3-dimethylaminopropyl)carbodiimide (EDC), N,N-diisopropylethylamine (DIPEA) and N-hydroxysuccinimide (NHS) were purchased from Sigma Aldrich (St. Louis, MO, USA). TUNEL assay kit and cathepsin B enzyme were purchased from R&D systems (Minneapolis, MN, USA). CellLight™ Lysosomes-RFP, BacMam 2.0 was purchased from Thermo Fisher Scientific Inc. (Rockford, IL, USA). TEM grid (Carbon Film 200 Mesh copper) was purchased from Electron Microscopy Sciences (Atlanta, GA, USA). DMEM media, fetal bovine serum (FBS), penicillin, and streptomycin were purchased from WELGENE Inc. (Daegu, Korea). The 4T1 (murine breast adenocarcinoma) and H9C2 (rat BDIX heart myoblast) cells were purchased from ATCC (American Type Culture Collection; Manassas, VA, USA).

### 2.2. Preparation and Characterization of FRRG-MMAE Nanoparticles

To prepare tumor-specific MMAE prodrug, the cathepsin B-specific cleavable FRRG (*Phe-Arg-Arg-Gly*) peptide was conjugated to the MMAE through a EDC/NHS one-step reaction. Briefly, FRRG peptide (200 mg, 1 eq), MMAE (219 mg, 1 eq), NHS (65 mg, 2 eq), EDC (106 mg, 2 eq) and DIPEA (2 eq) were dissolved in 10 mL DMF, followed by stirring at 37 °C for 24 h. Then, FRRG-MMAE was purified using reverse-phase high performance liquid chromatography (RP-HPLC; Agilent 1200 Series HPLC System, Santa Clara, CA, USA). The purity and exact molecular weight of FRRG-MMAE were characterized by RP-HPLC and matrix-assisted laser desorption/ionization time of flight (MALDI-TOF, AB Sciex TOF/TOF 5800 System, USA) mass spectrometer, respectively. The size distribution and particle morphology of FRRG-MMAE nanoparticles (1 mg/mL in saline) were analyzed using Zetasizer Nano ZS (Malvern Instruments, Worcestershire, UK) and transmission electron microscopy (TEM, CM-200, Philips, Bentonville, AR, USA), respectively. To assess the cathepsin B-specific cleavage of FRRG-MMAE nanoparticles, they were incubated with enzyme reaction buffer (MES buffer) containing 10 μg cathepsin B enzyme at 37 °C.

### 2.3. Cellular Uptake

The cellular uptake of FRRG-MMAE nanoparticles was assessed in the 4T1 cells. Briefly, 1 × 10^6^ 4T1 cells were seeded in the confocal dishes, followed by incubation with FRRG-MMAE (1 nM) at 37 °C. The tubulin formation after FRRG-MMAE nanoparticles or free MMAE treatments were visualized by staining with Cy5.5 fluorescent dye-conjugated anti-tubulin antibody for 24 h at 4 °C. Then, cells were fixed with 4% paraformaldehyde fixative for 25 min, and stained with DAPI solution (Invitrogen, Carlsbad, CA, USA) for 15 min in the dark. Finally, the 4T1 cells were observed using a confocal laser scanning microscope (CLSM) equipped with 405 diode (405 nm) and HeNe-Red (633 nm) lasers (Leica, Wetzlar, Germany).

### 2.4. Cytotoxicity Study

The cytotoxicity of FRRG-MMAE nanoparticles was assessed by cell counting kit-8 (CCK-8) assays. First, 5 × 10^5^ 4T1 or H9C2 cells were seeded in the 96-well cell culture plates. Then, the FRRG-MMAE nanoparticles or free MMAE were incubation with the cells for 24 h, followed by additional incubation with cell culture medium containing CCK-8 (10%) for 10 min. Finally, the cell viability of each cell was measured using a microplate reader with 450 nm of wavelength (VERSAmaxTM; Molecular Devices Corp., San Jose, CA, USA).

### 2.5. Antitumor Efficacy and Toxicity Study of FRRG-MMAE Nanoparticles in Breast Tumor Models

The antitumor efficacy was evaluated in the breast tumor models. The breast tumor models were prepared via subcutaneous injection of 1 × 10^6^ 4T1 cells. When the tumor volumes were approximately 100 mm^3^, mice were divided into three groups: (i) saline; (ii) MMAE (0.2 mg/kg); and (iii) FRRG-MMAE nanoparticles (equivalent dose of 0.2 mg/kg based on MMAE contents). The mice were treated once every three days, and tumor volumes were calculated as the smallest diameter^2^ × largest diameter × 0.53. The mice with a tumor volume of 2000 mm^3^ or larger were counted as dead. The toxicity study of FRRG-MMAE nanoparticles was assessed via histology. The major organs were collected from mice on day 5 after treatments, and structural abnormality in the organ tissues was assessed by staining with H&E.

### 2.6. Statistics

The statistical significance between two groups was analyzed using Student’s *t*-test. One-way analysis of variance (ANOVA) was performed for comparisons of more than two groups, and multiple comparisons were analyzed using the Tukey–Kramer post hoc test. Survival data were plotted as Kaplan–Meier curves and analyzed using the log-rank test. The statistical significance was indicated with asterisks (* *p* < 0.05, ** *p* < 0.01, *** *p* < 0.001) in the figures.

## 3. Results

### 3.1. Preparation and Characterization of FRRG-MMAE Nanoparticles

**Figure 2:**
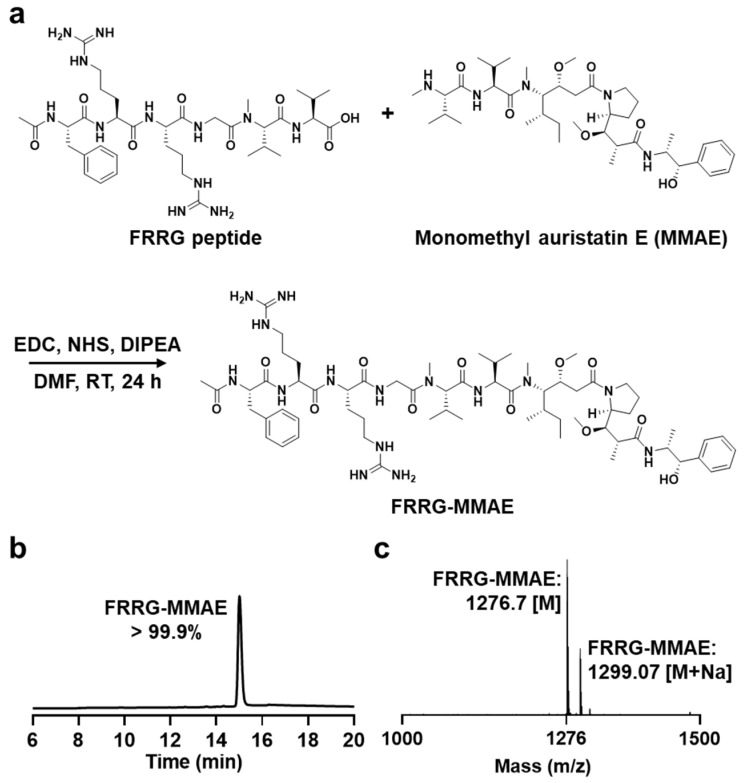
Preparation of FRRG-MMAE nanoparticles. (**a**) Synthetic route for preparation of tumor-specific monomethyl auristatin E (MMAE) prodrug nanoparticles, FRRG-MMAE. The (**b**) purity and (**c**) molecular weight of FRRG-MMAE after preparation.

The tumor-specific MMAE prodrug nanoparticles, FRRG-MMAE, constructed with cathepsin B-specific cleavable FRRG (*Phe-Arg-Arg-Gly*) peptide and MMAE, were designed to reduce the severe MMAE-related toxicity and to enhance their antitumor efficacy. The MMAE was chemically conjugated to the C-terminus of the FRRG peptide via one step EDC/NHS reaction (Figure 2a). After the preparation, 99.9% of FRRG-MMAE was purified with HPLC (Figure 2b). The successful synthesis was also confirmed using the MALDI-TOF mass spectrometer. The exact molecular weight of FRRG-MMAE was calculated to be 1276.64 Da for C_64_H_105_N_15_O_12_, and measured to be 1276.7 *m*/*z* [M] and 1299.07 [M+Na] (Figure 2c).

**Figure 3:**
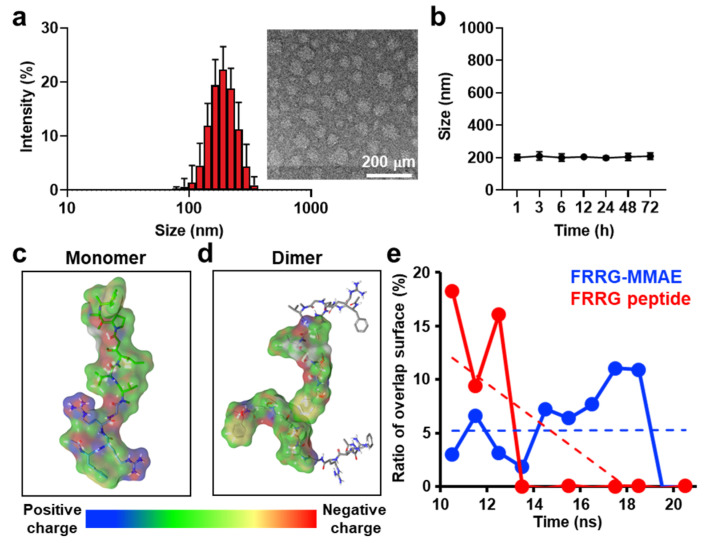

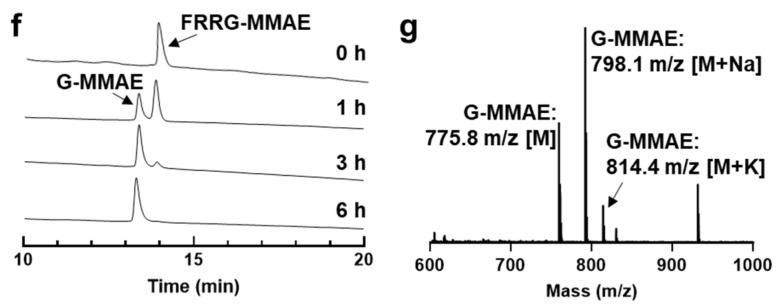
Characterization of FRRG-MMAE nanoparticles. (**a**) Size distribution and morphology of FRRG-MMAE nanoparticles. (**b**) The stability of FRRG-MMAE nanoparticles in mouse serum. (**c**,**d**) MD simulation results of (**c**) one or (**d**) two FRRG-MMAE molecules. (**e**) The ratio of overlap surface over total surface areas of the FRRG-MMAE of FRRG peptide molecules. (**f**) Enzymatic cleavage assays of FRRG-MMAE nanoparticles after incubation with cathepsin B. (**g**) Metabolite assay of FRRG-MMAE nanoparticles after incubation with cathepsin B.

Importantly, FRRG-MMAE molecules self-assembled into about 200 nm-sized spherical nanoparticles in aqueous condition without any additional carrier materials (Figure 3a). In addition, FRRG-MMAE nanoparticles were highly stable in the mouse plasma of physiological condition without significant changes of the particle size (Figure 3b). These stable structures with around 200 nm are suitable to accumulate within the tumor tissues via EPR effect [30]. The mechanism for the self-assembly of FRRG-MMAE molecules were assessed via molecular dynamic (MD) simulation. The MD simulation results of one FRRG-MMAE molecule showed the amphiphilic characteristics due to the positively charged polar arginine sequences (blue color) and neutrally charged non-polar MMAE molecules (Figure 3c and Figure S1). In addition, MD simulation results of two FRRG-MMAE molecules indicated that MMAE in each FRRG-MMAE maintain a close distance for 20 ns, whereas the distance between two FRRG (without MMAE) molecules became distant because of electrostatic repulsion in the side chains (Figure 3d and Figure S2). Similar results were also observed in the MD simulation results, which are performed with ten FRRG-MMAE molecules (Figure S3). As a result, the ratio of overlap surface over total surface areas of the FRRG-MMAE molecules was sustainably maintained, while that of the FRRG peptide converged to zero (Figure 3e). These results clearly demonstrate that the self-assembly mechanism of FRRG-MMAE molecules are hydrophobic interactions due to the MMAE molecules [29]. Next, the target-enzyme specificity of FRRG-MMAE nanoparticles was evaluated by incubation with cathepsin B. When the FRRG-MMAE nanoparticles were incubated with cathepsin B in the MES buffer (pH 5.5), they began to release the glycine-conjugated MMAE (G-MMAE) from 1 h of incubation (Figure 3f). In addition, over 90% of FRRG-MMAE nanoparticles cleaved to G-MMAE after 3 h of incubation and eventually fully cleaved 6 h post-incubation. This result was supported by MALDI-TOF analysis, which verify the exact molecular weight of G-MMAE (calculated mass: 775.58 Da, measured mass: 775.8 *m*/*z* [M], 798.1 [M+Na], and 814.4 [M+K]) at a newly appeared characteristics peak in the HPLC spectrum (approximately 13 min) after incubation of FRRG-MMAE nanoparticles with cathepsin B (Figure 3g). It was previously studied that glycine (G)-conjugated drug is subsequently metabolized into the free drug by intracellular proteases [14]. Taken together, these results demonstrate that FRRG-MMAE nanoparticles offer simple synthetic chemistry for industrial-scale manufacturing and form stable nanoparticles that specifically release MMAE molecules by cathepsin B-mediated enzymatic cleavage.

### 3.2. Cellular Uptake and Tumor Cell Selective Cytotoxicity of FRRG-MMAE Nanoparticles

**Figure 4:**
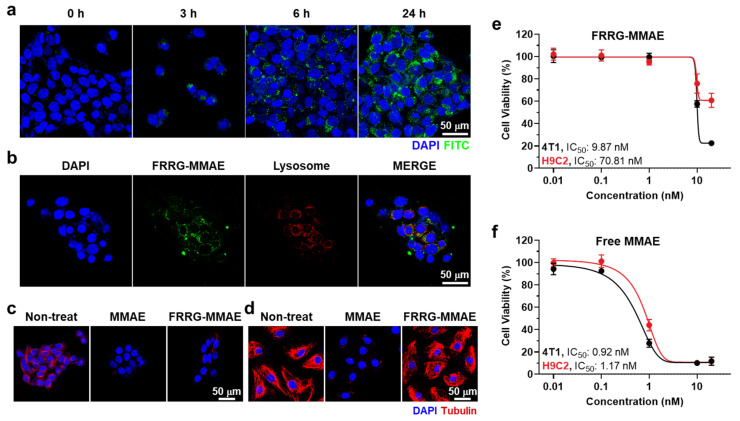
Cellular uptake and tumor cell selective cytotoxicity of FRRG-MMAE nanoparticles. (**a**) Cellular uptake of FRRG-MMAE nanoparticles in 4T1 cells. (**b**) Co-localization of FRRG-MMAE nanoparticles and lysosomes in 4T1 cells. (**c**,**d**) The tubulin formation of (**c**) 4T1 and (**d**) H9C2 cells after treatment with free MMAE or FRRG-MMAE. (**e**,**f**) The cell viability of (**e**) 4T1 and (**f**) H9C2 cells after treatment with free MMAE or FRRG-MMAE.

The cellular uptake of FRRG-MMAE nanoparticles was assessed in the murine breast carcinoma (4T1) cells. For the efficient monitoring, FRRG-MMAE nanoparticles containing 10% of FITC fluorescent dye-conjugated FRRG-MMAE were used in the experiments. When the 4T1 cells were treated with FRRG-MMAE nanoparticles (1 nM) at 37 °C, a robust uptake was clearly observed, wherein the FITC fluorescence signals of nanoparticles were gradually increased in the cells with an incubation time-dependent manner (Figure 4a). We also observed strong co-localization of FRRG-MMAE nanoparticles with lysosomes in the 4T1 cells after 24 h of incubation (Figure 4b). These results clearly indicate that FRRG-MMAE nanoparticles internalize in the cells via the nanoparticle-derived endosomal-lysosomal endocytosis mechanism. Next, the selective MMAE release of FRRG-MMAE nanoparticles by cathepsin B was evaluated in 4T1 cells and rat BDIX cardiomyocytes (H9C2). This is because 4T1 cells express significantly high levels (32.48 ± 3.14-fold) of cathepsin B than H9C2 cells [28]. The tubulin formation in the 4T1 cells was significantly inhibited after 24 h of treatment with free MMAE or FRRG-MMAE nanoparticles compared to naive cells (Figure 4c). Importantly, the effect to inhibit tubulin formation in the cells of free MMAE was similar in the H9C2 and 4T1 cells, whereas FRRG-MMAE nanoparticle-treated H9C2 cells showed similar tubulin formation with naive cells (Figure 4d). These results indicate that FRRG-MMAE nanoparticles inhibit tubulin polymerization by releasing MMAE molecules owing to the overexpressed cathepsin B in the tumor cells, but they maintain inactive states in the cathepsin B-deficient normal cells. In agreement with these results, the IC_50_ value of FRRG-MMAE nanoparticles in the 4T1 cells was measured to be 9.87 nM after 24 h incubation, while it was 70.81 nM in the H9C2 cells, which showed an approximately seven-fold difference, indicating tumor cell selective cytotoxicity (Figure 4e). In contrast, free MMAE showed indiscriminate cytotoxicity in both cells with nearly similar IC_50_ values (Figure 4f). These results indicate that FRRG-MMAE nanoparticles can efficiently eradiate the tumors with reduced MMAE-related severe toxicity toward normal tissues by tumor cell selective cytotoxicity.

### 3.3. Antitumor Efficacy and Safety of FRRG-MMAE Nanoparticles in Breast Tumor-Bearing Mice

**Figure 5:**
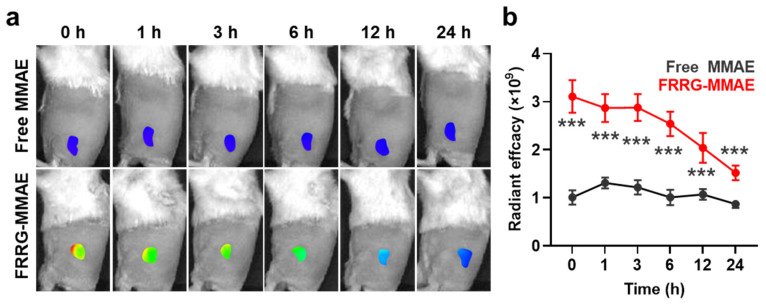
Tumor targeting of FRRG-MMAE nanoparticles. (**a**) NIRF images of 4T1 tumor-bearing mice treated with fluorescent dye Cy5.5-conjugated free MMAE or FRRG-MMAE. (**b**) Quantitative analysis for fluorescence intensities in the tumor regions of NIRF images. The statistical significance was indicated with asterisks *** *p* < 0.001 in the figures.

The high tumor targeting of FRRG-MMAE nanoparticles via the EPR effect was assessed in the breast tumor-bearing mice that were established by subcutaneous inoculation of 4T1 cells into the flank of the BALB/C mice. Fluorescent dye Cy5.5-conjugated free MMAE or FRRG-MMAE with an equivalent dose of 0.2 mg/kg of MMAE content were intravenously injected in the mice. The NIRF images of the 4T1 tumor-bearing mice showed that a significantly higher number of FRRG-MMAE nanoparticles was accumulated in the targeted tumor tissues compared to free MMAE (Figure 5a). Quantitatively, a 2.98–3.01-fold higher number of FRRG-MMAE nanoparticles accumulated within the tumor tissues compared to free MMAE (Figure 5b).

**Figure 6:**
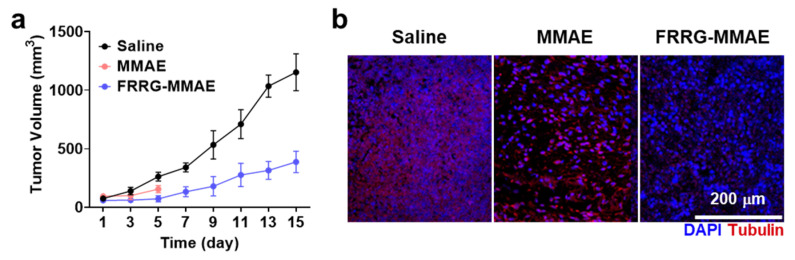

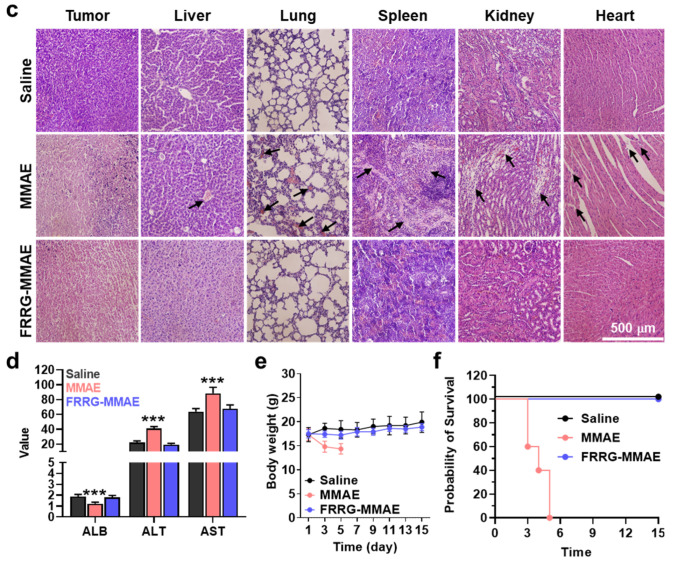
Antitumor efficacy and safety of FRRG-MMAE nanoparticles in breast tumor-bearing mice. (**a**) Tumor growth of 4T1 tumor-bearing mice during treatment with free MMAE or FRRG-MMAE nanoparticles once every three days. (**b**) Tumor tissues stained with fluorescent dye Cy5.5-conjugated tubulin antibody after 5 days of treatment. (**c**) Tumor and organ tissues stained with H&E after 5 days of treatment with free MMAE or FRRG-MMAE nanoparticles. (**d**) Hematological parameters of mice treated with saline, free MMAE or FRRG-MMAE for 5 days. (**e**) The body weight of mice during treatment with free MMAE or FRRG-MMAE nanoparticles once every three days. (**f**) The mice survival. The statistical significance was indicated with asterisks *** *p* < 0.001 in the figures.

To evaluate the antitumor efficacy and safety of FRRG-MMAE nanoparticle treatment, 4T1 tumor-bearing mice were treated with free MMAE (0.2 mg/kg) or FRRG-MMAE nanoparticles (equivalent dose of 0.2 mg/kg based on MMAE contents) once every three days. As expected, FRRG-MMAE nanoparticles (71.92 ± 26.03 mm^3^) significantly inhibited the tumor growth compared to saline (261.81 ± 37.33 mm^3^; *p* < 0.001) and free MMAE (154.73 ± 31.63 mm^3^; *p* < 0.001) groups on day 5 after treatment (Figure 6a). In addition, tumor tissues stained with fluorescent dye Cy5.5-conjugated tubulin antibody showed a significantly inhibited tubulin formation compared to saline and free MMAE groups after five days of treatment (Figure 6b). Tumor tissues stained with H&E also showed greatly elevated structural abnormalities from the extensive tumor areas in the FRRG-MMAE nanoparticle group than saline and free MMAE groups after five days of treatments (Figure 6c). Quantitatively, the ratio of the damaged areas in the tumor tissues from mice treated with FRRG-MMAE nanoparticles were 14.31–14.48-fold and 1.63–1.88-fold higher compared to those treated with saline and free MMAE, respectively (Figure S4). This is attributable to the high tumor accumulation owing to the EPR effect by 200 nm-sized stable structure of FRRG-MMAE nanoparticles [30]. To directly compare the antitumor efficacy of free MMAE and FRRG-MMAE nanoparticles for 15 days, tumor growth of 4T1 tumor-bearing mice was additionally assessed during treatment with 0.1 mg/kg MMAE once every three days. This is because mice treated with 0.2 mg/kg MMAE once every three days were all dead within five days owing to their severe systemic toxicity. The results demonstrated that free MMAE-treated mice showed rapid tumor growth compared to FRRG-MMAE-treated mice during monitoring for 15 days (Figure S5). The safety of FRRG-MMAE nanoparticle treatment was evaluated via H&E staining of normal organs (liver, lung, spleen, kidney and heart) after five days of treatment. As shown in Figure 6c, mice treated with free MMAE showed severe systemic toxicity, wherein the structural abnormalities (black arrows) were clearly observed in all the organs. Moreover, free MMAE-treated mice showed significant changes in the hematological parameters related with liver or kidney toxicities including albumin (ALB), aspartate aminotransferase (AST) and alanine transaminase (ALT), whereas those in the mice treated with FRRG-MMAE nanoparticles were in normal range and similar with saline group after five days of treatment (Figure 6d). Finally, mice treated with free MMAE showed significant body weight loss owing to the severe toxicity; eventually, all the mice were dead within five days of treatment (Figure 6e,f). In contrast, FRRG-MMAE nanoparticle-treated mice exhibited no significant changes in the body weight compared to saline group, wherein the no death cases occurred during 15 days of treatment. These results show the significantly reduced MMAE-related toxicity in the FRRG-MMAE nanoparticle group. Collectively, our findings demonstrate that FRRG-MMAE nanoparticles effectively inhibit the tumor growth, owing to the tumor-specific MMAE release and the high tumor accumulation due to the nanoparticle-derived EPR effect, as well as reduce MMAE-related toxicity by high cathepsin B-specificity, thereby allowing safe and effective chemotherapy.

## 4. Conclusions

In summary, we proposed tumor-specific MMAE prodrug nanoparticles for safe and effective chemotherapy. FRRG-MMAE, which consists of cathepsin B-specific cleavable FRRG peptide and MMAE, formed stable nanoparticles without any additional carrier materials by hydrophobic interaction-derived aggregations. Importantly, highly accumulated FRRG-MMAE nanoparticles in the tumor tissues via the EPR effect selectively released MMAE molecules in cathepsin B-overexpressed tumor cells, which induced a potent antitumor efficacy by inhibiting tubulin polymerization. Meanwhile, FRRG-MMAE nanoparticles significantly minimized the MMAE-related toxicity toward normal tissues owing to their innately low cathepsin B. As a result, efficient tumor delivery of MMAE by FRRG-MMAE nanoparticles greatly inhibited the breast tumor growth with minimal side effects. Compared with the conventional drug delivery system encapsulating anticancer drugs into the nanoparticles, this carrier-free prodrug nanoparticle system can mitigate the potential off-target toxicity by carrier materials. In addition, its precise and concise structures can achieve the simple one step synthetic protocol, thereby overcoming the formidable challenges of nanoparticles for clinical translation, such as difficulty in industrial-scale manufacturing and quality control (QC). Therefore, this study offers a promising approach for safe and effective chemotherapy via MMAE-based prodrug nanoparticles, which may open new avenues for advanced drug design for translational nanomedicine.

## Data Availability

All relevant data are available with the article and its supplementary information files, or available the corresponding authors upon reasonable requests.

## Supplementary Materials

The following supporting information can be downloaded at: https://www.mdpi.com/article/10.3390/pharmaceutics14102131/s1, Figure S1: Molecular dynamic (MD) simulation of one FRRG-MMAE molecule. Figure S2: Molecular dynamic (MD) simulation of two (a) FRRG-MMAE or (b) FRRG peptide molecules. Figure S3: Molecular dynamic (MD) simulation of ten molecules of (a) FRRG-MMAE or (b) FRRG peptide. The system was fully relaxed for 50 ns due to large number of molecules, and the snapshot images from 50 to 100 ns in simulation time were used. The images showed that the aggregation was driven by the intermolecular interaction in MMAE segments, whereas the FRRG peptides were separated without significant aggregations. Figure S4: The percentages of apoptosis area in tumor tissues after 5 days of treatment with free MMAE or FRRG-MMAE nanoparticles. Figure S5: Tumor growth of 4T1 tumor-bearing mice during treatment with 0.1 mg/kg MMAE once every three days.
