# Supplementary material for: Tumor-Specific Monomethyl Auristatin E (MMAE) Prodrug Nanoparticles for Safe and Effective Chemotherapy
Source: Pharmaceutics. 2022 Oct 7;14(10):2131. doi: 10.3390/pharmaceutics14102131 (PMC9609178; doi:10.3390/pharmaceutics14102131)
Supplement: Supplementary file 1 [file pharmaceutics-14-02131-s001.zip › pharmaceutics-1870608-supplementary.pdf]

Supplementary Materials: Tumor-specific monomethyl auristatin E (MMAE) prodrug nanoparticles for safe and effective chemotherapy

Hanhee Cho, Man Kyu Shim, Yujeong Moon, Sukyung Song, Jinseong Kim, Jiwoong Choi, Jeongrae Kim, Jung Yeon Park, Yongju Kim, Cheol-Hee Ahn, Hong Yeol Yoon, Kwangmeyung Kim

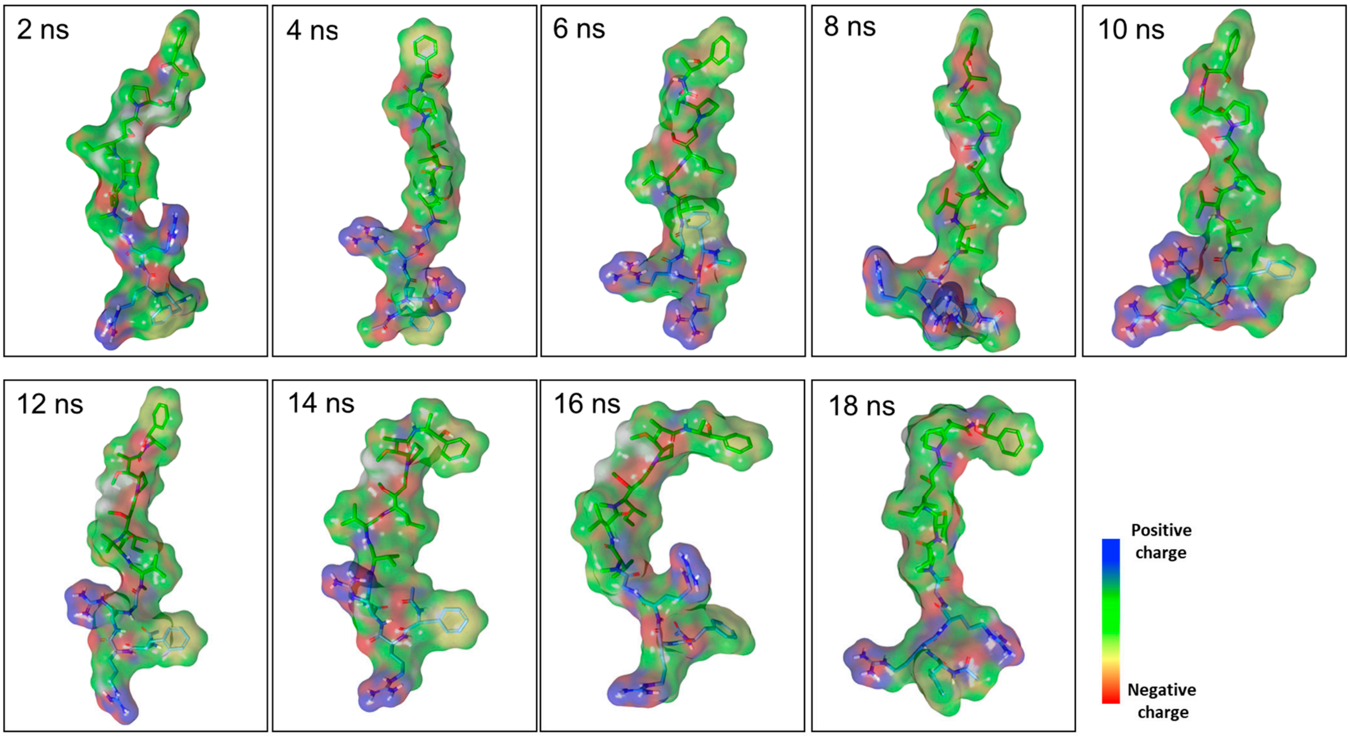

Figure S1. Molecular dynamic (MD) simulation of one FRRG-MMAE molecule.

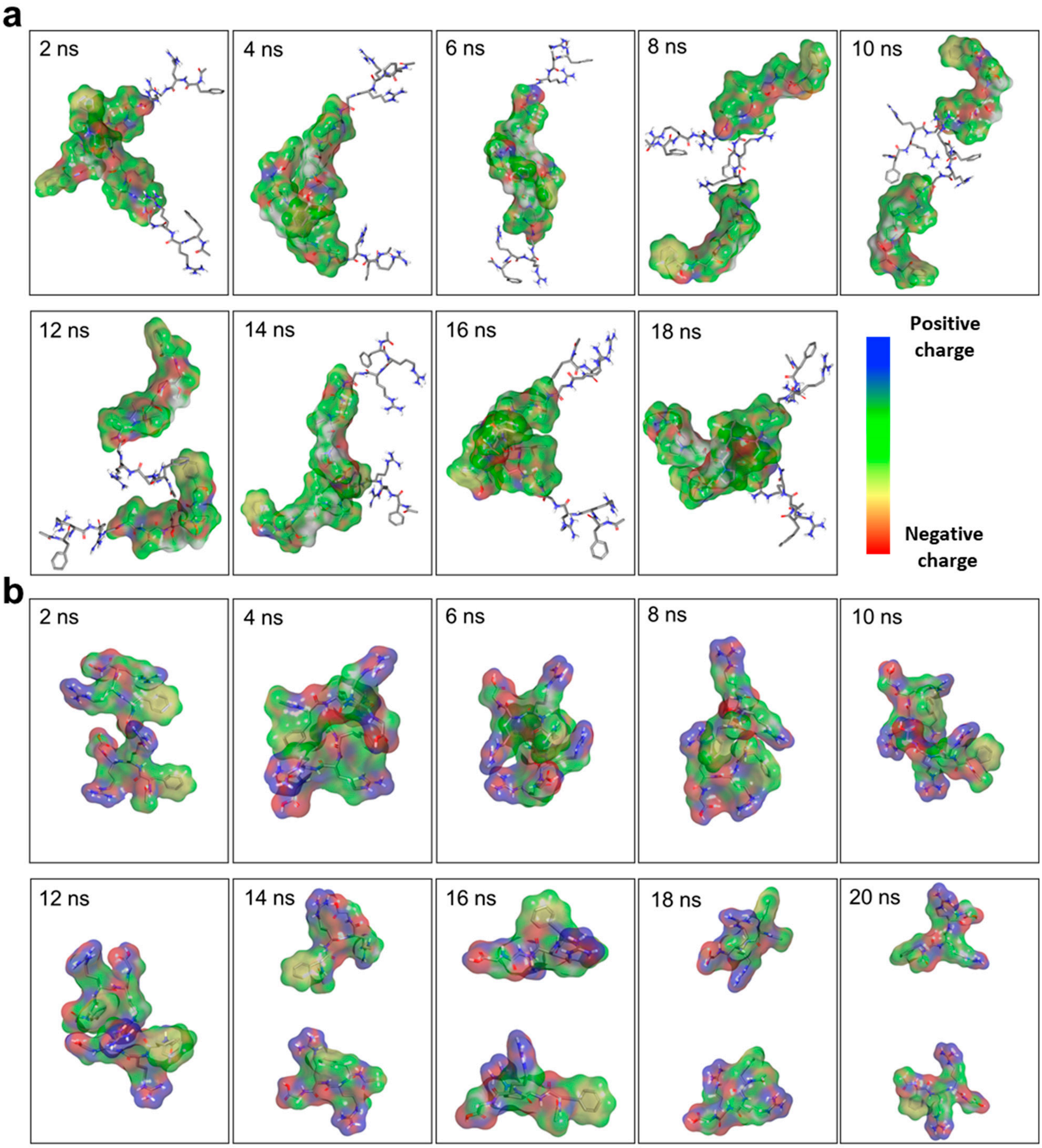

Figure S2. Molecular dynamic (MD) simulation of two (a) FRRG-MMAE or (b) FRRG peptide molecules.

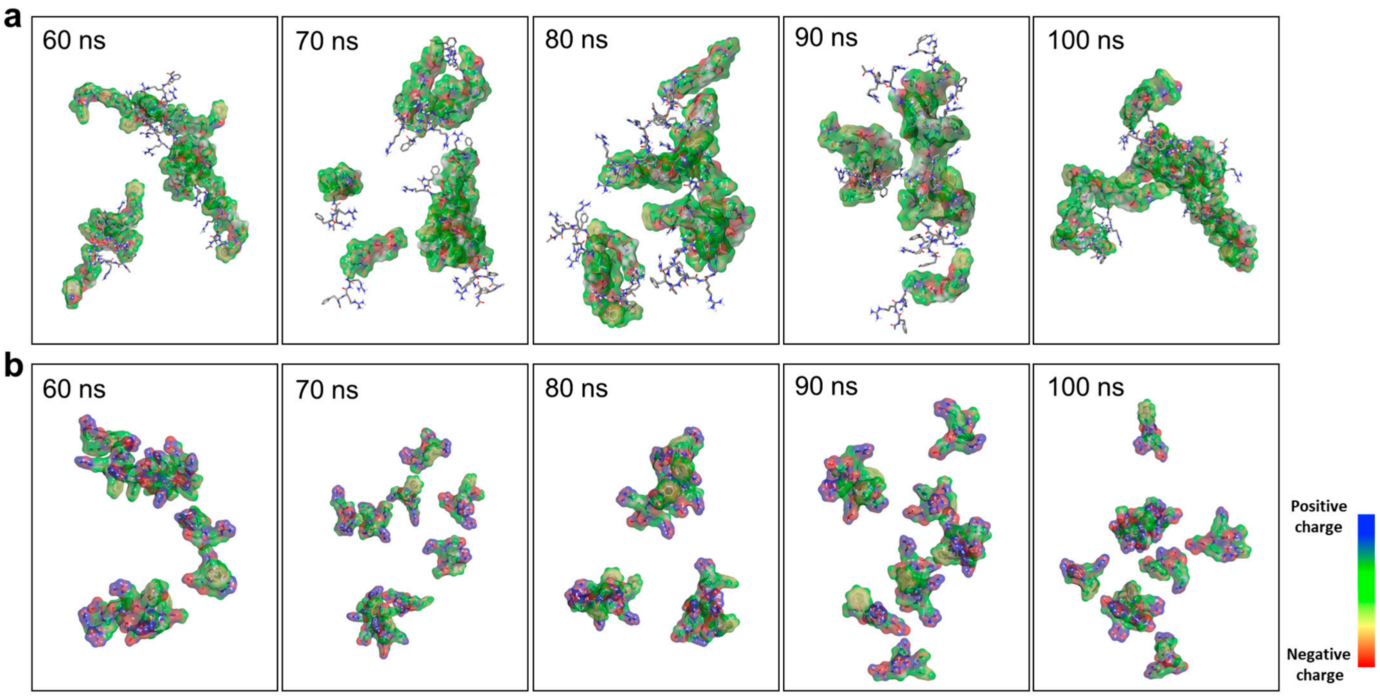

Figure S3. Molecular dynamic (MD) simulation of ten molecules of (a) FRRG-MMAE or (b) FRRG peptide. The system was fully relaxed for 50 ns due to large number of molecules, and the snapshot images from 50 to 100 ns in simulation time were used. The images showed that the aggregation was driven by the intermolecular interaction in MMAE segments, whereas the FRRG peptides were separated without significant aggregations.

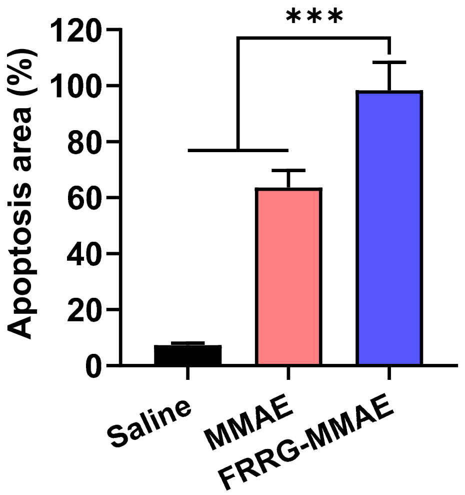

Figure S4. The percentages of apoptosis area in tumor tissues after 5 days of treatment with free MMAE or FRRG-MMAE nanoparticles. *** $p < 0.001$.

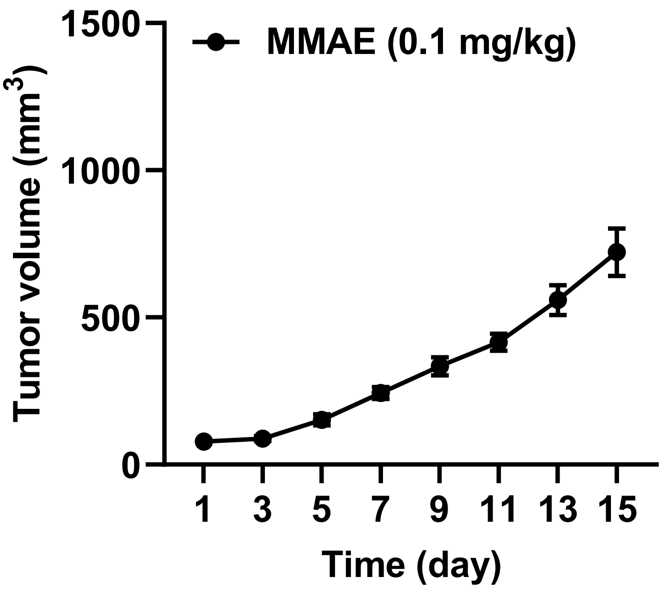

Figure S5. Tumor growth of 4T1 tumor-bearing mice during treatment with 0.1 mg/kg MMAE once every three days.
